# Rapid Detection of *Streptococcus mutans* Using an Integrated Microfluidic System with Loop-Mediated Isothermal Amplification

**DOI:** 10.4014/jmb.2304.04026

**Published:** 2023-05-19

**Authors:** Jingfu Wang, Jingyi Wang, Xin Chang, Jin Shang, Yuehui Wang, Qin Ma, Liangliang Shen

**Affiliations:** 1 State Key Laboratory of Military Stomatology and National Clinical Research Center for Oral Diseases & Shaanxi Engineering Research Center for Dental Materials and Advanced Manufacture, Department of Cranio-facial Trauma and Orthognathic Surgery, School of Stomatology, The Fourth Military Medical University, Xi'an 710032, P.R.China; 2 Department of Stomatology, General Hospital of Northern Theater Command, 83 Wenhua Road, Shenyang 110016, P.R.China; 3 College of Information and Electrical Engineering, Shenyang Agricultural University, Shenyang 110866, P.R.China; 4 Outpatient Department, The Ninth Retired Cadres Retreat of Liaoning Military Command, 176 Dongbei Road, Shenyang 110044, P.R.China; 5 Department of Biochemistry and Molecular Biology, Fourth Military Medical University, Xi'an, P.R.China

**Keywords:** *Streptococcus mutans*, biosensor, loop-mediated isothermal amplification, rapid detection, dental caries

## Abstract

*Streptococcus mutans* is the primary causative agent of caries, which is one of the most common human diseases. Thus, rapid and early detection of cariogenic bacteria is critical for its prevention. This study investigated the combination of loop-mediated isothermal amplification (LAMP) and microfluid technology to quantitatively detect *S. mutans*. A low-cost, rapid microfluidic chip using LAMP technology was developed to amplify and detect bacteria at 2.2–2.2 × 10^6^ colony-forming units (CFU)/ml and its detection limits were compared to those of standard polymerase chain reaction. A visualization system was established to quantitatively determine the experimental results, and a functional relationship between the bacterial concentration and quantitative results was established. The detection limit of *S. mutans* using this microfluidic chip was 2.2 CFU/ml, which was lower than that of the standard approach. After quantification, the experimental results showed a good linear relationship with the concentration of *S. mutans*, thereby confirming the effectiveness and accuracy of the custom-made integrated LAMP microfluidic system for the detection of *S. mutans*. The microfluidic system described herein may represent a promising simple detection method for the specific and rapid testing of individuals at risk of caries.

## Introduction

Caries is a bacterial disease with a high prevalence and widespread distribution, affecting more than one-third of the global population and 60–90% of school-aged children [1]. Thus, caries is a common oral condition and one of the most common illnesses in humans [2]. Along with cancer and cardiovascular illnesses, dental caries has been identified by the World Health Organization as one of the three major diseases that can be prevented and cured [3]. Caries can cause tooth defects and lead to tooth loss if left untreated, which seriously affects quality of life. Therefore, wide-range general surveys should be conducted to promote its early diagnosis and prevention.

Bacteria form biofilms by adhering to and aggregating on the tooth surface, which produce acid and break down carbohydrates. Indeed, biofilm formation by microorganisms is considered to be the initiating factor of caries. The gram-positive bacterium *Streptococcus mutans* is the main constituent of dental plaque biofilms and is thus considered to be responsible for tooth decay [4]. Early-stage caries is difficult to observe, although it can contain up to 10-times the number of *S. mutans* in healthy plaques. Histopathology, microbiology [5], immunohistochemistry [6], and molecular techniques [7] are commonly used for detecting *S. mutans*. In particular, nucleic acid amplification through polymerase chain reaction (PCR) is widely used to detect pathogens and plays an important role in laboratory confirmation owing to its high precision and specificity [8, 9]. Indeed, PCR is regarded as the “gold standard” for detecting the presence of disease-causing pathogens [10]; however, its complexity and the necessity for advanced equipment prevent it from being widely applied in outpatient clinics and private dentistry offices. Hence, better, rapid, precise, sensitive, and specific tools to detect *S. mutans* are urgently needed for monitoring and preventing caries progression.

Since it was first reported, the isothermal amplification technology has been widely used to enhance nucleic acid detection for different applications [11-14]. In particular, loop-mediated isothermal amplification (LAMP) is a simple, rapid, and specific method for nucleic acid amplification [15]. It employs a DNA polymerase from *Bacillus stearothermophilus* that has cycling-strand displacement DNA activity [16] and primers designed to target specific parts of a gene, the gene products are then exposed to isothermal conditions to form a loop-structure product, so that the target gene can be amplified efficiently, quickly, and specifically [17, 18]. Compared with conventional PCR, LAMP does not require template thermal denaturation or temperature cycling; therefore, it is less time-consuming. This technology is comparable to or even better than PCR, in terms of sensitivity, specificity, detection range, and other parameters [19, 20]. Moreover, the cost is equal to or even lower than that of PCR. Hence, LAMP is a promising technique for rapid and on-site detection of cariogenic bacteria. Despite the aforementioned benefits, the LAMP technology still requires professional laboratory personnel and equipment; thus, there are still some restrictions on the capability of this technology to rapidly detect pathogens in medical settings, which may be overcome by recent advancements in microfluidic technologies.

A microfluidic system is an automated operating system composed of micropumps, valves, drainpipes, and channels for content analysis [21]. Microfluidic systems can combine various detection techniques, such as sample processing, reagent handling, bioreaction, and detection, on a single platform. These techniques are characterized by rapid detection, ease of use, cost-effectiveness, lack of expensive equipment and specialized personnel, lack of contamination risk, and high accuracy of pathogen detection [22, 23]. The miniaturized microfluidic technology has been rapidly developing since it was first described by Terry *et al*. in 1979 [24], particularly for pathogen detection. Currently, this technology is considered a complementary method for diagnostic procedures in the setting of infectious diseases [25]. Noteworthily, the combination of LAMP and microfluidic technology can maximize their merits and minimize their flaws. Fang *et al*. developed a microfluidic chip with integrated LAMP that directly analyzed 0.4 μl of DNA in less than 1 h, with a detection limit of 10 fg/μl for *Pseudorabies virus* [26]. Nguyen *et al*. reported a sample-in-result-out device for the automatic detection of three pathogenic bacteria (*Escherichia coli* O157:H7, *Salmonella typhimurium*, and *Vibrio parahaemolyticus*) using LAMP on a centrifugal disc within 1 h and with a low detection limit of 10^2^ cells/ml [27]. Nevertheless, the microfluidic technology has not been widely applied in stomatology, being mainly employed to detect or simulate mucous membrane illnesses and tumors; moreover, it is less frequently used for the rapid detection of microorganisms. The microfluidic technology was first applied for oral disease diagnosis in 2007 [28]. Li *et al*. used a microfluidic chip for rapid DNA amplification and onsite detection of three oral pathogens (*Porphyromonas gingivalis*, *Treponema denticola*, and *Tannerela forsythia*), with significantly shortened detection time and a detection limit of 125 colony-forming units (CFU)/μl [29]. Of note, to the best of our knowledge, microfluidic devices to detect causative microorganisms of dental caries have not been reported to date.

In this study, a LAMP-based microfluidic detection platform for the rapid and quantitative detection of *S. mutans* was developed. To the best of our knowledge, this is the first proposed LAMP-based microfluidic platform for *S. mutans*. Our pathogen detection platform is expected to eliminate the traditional tedious manual operation steps and the possibility of contamination, simplify detection conditions, and shorten detection time in the clinical setting. This device may represent a promising point-of-care testing tool for *S. mutans*, which can be extended for the detection of other cariogenic bacteria.

## Materials and Methods

### Chemicals and Reagents

LAMP amplification and detection reagents were provided by HaiGene Biotech (China). LAMP primers were synthesized by Sangon Biotech (China). The lysis buffer for microorganisms was purchased from Takara Bio (Japan).

### Bacterial Culture and CFU Test

*S. mutans* UA159 strain was provided by Jilin University (China). *Porphyromonas gingivalis* (*P. gingivalis*), *Porphyromonas endodontalis* (*P. endodontalis*), and *Enterococcus faecalis* (*E. faecalis*) were provided by China Medical University (China). The *S. mutans* were inoculated on brain–heart infusion agar and cultured in an anaerobic environment at 34°C for 48 h. The other 3 bacteria were inoculated on blood agar and cultured in an anaerobic environment at 37°C for 48 h. Subsequently, gram staining and identification of the bacteria were performed under a microscope (Fig. S1). Finally, the colonies in the plate were photographed and counted, and the average number of three replicate plates was recorded.

### Primer Design

DNA sequences of the target genes were obtained from the National Center for Biotechnology Information (http://www.ncbi.nlm.nih.gov). The gene sequences of *dexA*, *gtfB*, and *spaP* of *S. mutans* were selected as alternative sequences. Primer Explore V5 online LAMP primer design software (Eiken Chemicals Co., Japan) was used for primer design. One set of primers was chosen from 43 sets of LAMP primer schemes that were screened using BLAST (https://blast.ncbi.nlm.nih.gov/Blast.cgi).

### Microfluidic Chip Design and Fabrication

**Fig. 1:**
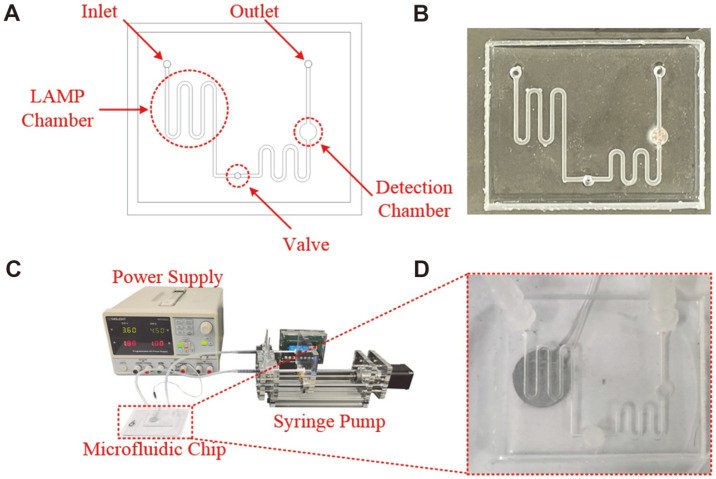
Construction of the microfluidic detection system. (**A**) Schematic diagram of the microfluidic chip. (**B**) Photograph of the microfluidic chip. (**C**) Photograph of the actuation system. (**D**) A PTC ceramic heater was placed under the serpentine channel of the microfluidic chip.

A schematic diagram of the microfluidic chip is shown in Fig. 1A. The chip had a size of 50 × 40 × 5 mm and included a serpentine channel (height × width, 1 × 0.8 mm) for the LAMP reaction, a valve, and a detection chamber (height × diameter, 1 × 5 mm) (21 μl lyophilized orange-green dye preloaded) to monitor the color (Fig. 1B).

**Fig. 2:**
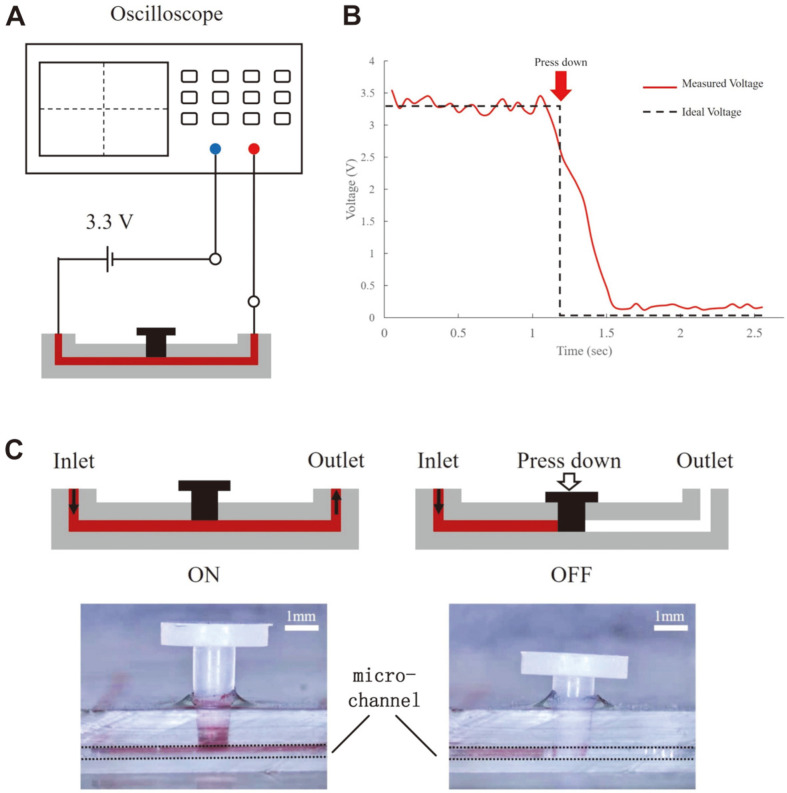
Capacity of the valve in the microfluidic detection system. (**A**) Schematic of the system used to test the microvalve. The inlet and outlet of the chip were connected to the positive pole of a power supply (3.3 V constant voltage) and an oscilloscope, respectively. When the valve was open, the KCl solution formed a loop with the power supply system and the voltage was measured by the oscilloscope. When the valve was closed, the solution was blocked, the loop was broken, and the voltage value became 0. (**B**) Schematic and micrograph of the ON and OFF state of the valve. When the valve was opened, the magenta solution passed through and filled the microchannel. When the valve was closed, the magenta solution was blocked, and the red solution was detected in the downstream portion of the microchannel. Scale bar = 1 mm. (**C**) Voltage variation when the plug was ON and OFF. When the valve was pressed down, the voltage decreased to almost 0, which indicated the blocking capacity of the valve.

A positive temperature coefficient (PTC) ceramic heater (Sangni Electronics Co., China) was placed under the serpentine channel and heated to 65°C. The microfluidic chip was fabricated using a polydimethylsiloxane channel layer and a glass substrate using oxygen plasma bonding. The mold for the polydimethylsiloxane channel layer was first designed using SolidWorks software (Dassault Systèmes Solidworks Corp., USA) and then printed using a 3D printer (Form 3; FormLabs, USA). The elastomer and curing agent were mixed at a mass ratio of 10:1 and heated to 55°C for 4 h. The actuation system consisted of one microfluidic chip, one syringe pump, and one dual-output power supply (Figs. 1C and 1D). A dual-output power supply was used to provide voltage to the PTC ceramic heater. The syringe pump was set to push the mixture of the lysed sample and the LAMP reagent at a flow rate of 20 L/min. The Heat Transfer Module in COMSOL Multiphysics software (Comsol, USA) was used to simulate and analyze the heating process of the PTC heater. A moving plug was used as the valve in the microchannel to prevent backflow. Magenta and potassium chloride (KCl) solutions were used to evaluate the effects of the valve. The inlet and outlet of the microvalve chip were connected to the positive pole of the power supply (provided a 3.3 V constant voltage) and an oscilloscope, respectively (Fig. 2A). The oscilloscope recorded the voltage changes when the valve was opened and closed.

### Construction of the Detection System

**Fig. 3:**
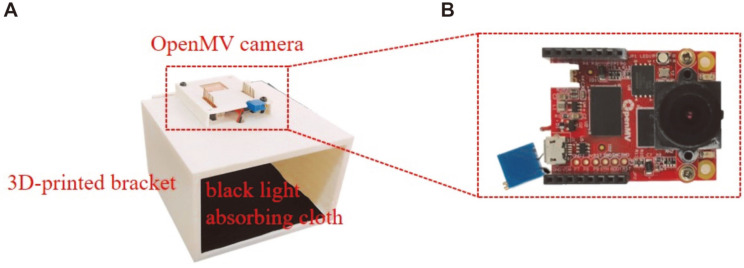
Construction of the detection system. (**A**) Photograph of the assembled detection system with a threedimensional- printed bracket, OpenMV camera, and black light-absorbing cloth. (**B**) Photograph of the OpenMV camera.

Python high-level scripts were used to program the OpenMV camera such that it possessed image acquisition and recognition functions. To guarantee the consistency of image capture and recognition of each result, the OpenMV camera was integrated into a 3D printed bracket (Fig. 3). A white light source and black light-absorbing fabric were inserted into the bracket to ensure consistency of the exterior circumstances throughout image acquisition and recognition.

### Benchtop LAMP Assay

For nucleic acid extraction, a simple lysis method was used. DNA lysis buffer (50 μl) and bacterial solution (2 μl) were mixed and incubated in an 80°C water bath for 15 min. For assay sensitivity, the bacterial solution was serially diluted 10-fold with double-distilled water (ddH_2_O) from 2.2 × 10^6^ to 2.2 CFU/ml. Each reaction was performed in triplicate. To test the specificity of the LAMP assay, three common oral bacteria were used-*P. gingivalis*, *P. endodontalis*, and *E. faecalis*—at a concentration of 10^8^ CFU/ml. RNase-free ddH_2_O was used as a negative control.

The LAMP reaction was performed in a 21 μl system containing 15 μl of LAMP OG reagent, 2 μl of LAMP primer mix, 2 μl of ddH_2_O, and 2 μl of lysed solution. The reaction was conducted in a 65°C water bath for 30 min. Afterward, the OG dye was added to the reaction, and the solution was gently mixed and shaken for 30 s. The tubes were then placed upright to observe the outcomes.

### LAMP-Based Microfluidic System Protocol

The lysed bacteria were placed in a LAMP chamber that had already been preloaded with the LAMP reaction mixture. A PTC ceramic heater was used to increase and maintain the temperature at 65°C for 30 min. At this stage, the valve was closed to ensure that the mixture was not pushed into the detection chamber, thereby terminating the reaction. When the LAMP reaction was complete, the valve was opened so that the reaction solution could be transported to the detection chamber, which was preloaded with OG dye using a syringe pump system. After mixing, the microfluidic chip was observed and analyzed using the OpenMV camera, which was programmed to automatically segment the colored samples, convert the color signal to L*a*b* (lightness, red/green, and blue/yellow) color values (which is a mode for measuring colors based on the Commission International Eclairage [30]), and calculate the average Lab value of the region. Finally, the signal data were collected.

### PCR Amplification

The PCR reaction system contained 2 μl of *S. mutans* genomic DNA, 10 μM of each primer (F3 and B3), 5 μl of 2 × Es Taq MasterMix (Cwbio, China), and 2 μl of ddH_2_O. Primers F3 and B3 were used to amplify the same specific region of *spaP* detected in the LAMP assay. The PCR amplification was performed as follows: initial denaturation at 94°C for 3 min; 28 cycles of 94°C for 30 s, 53°C for 30 s, and 72°C for 30 s; and final extension at 72°C for 2 min. After amplification, the PCR products were separated on a 1.0% agarose gel at 120 V for 30 min. DNA Marker DL2000 (Bioer, China) was used for size reference.

## Results and Discussion

### Primer Design and Target Sequence

**Fig. 4:**
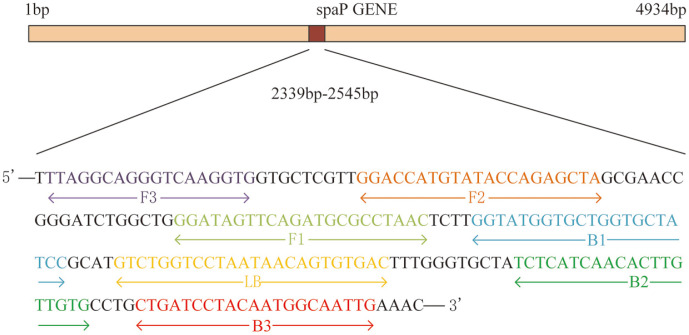
Schematic of the *Streptococcus mutans* spaP gene and the corresponding location of each LAMP primer.

Detection effectiveness is significantly influenced by the melting temperature of the primers and GC content of the target sequence. Noteworthily, the amplification reaction is favored in sequences with GC concentrations within 40–60%. Hence, a *spaP* sequence with a GC content of 40–60% was selected for further analysis (GenBank Accession No. APQ13_RS04955; from 2,339 to 2,545 bp), and the LAMP primers were designed accordingly (Fig. 4). LAMP is usually more specific than conventional PCR because it employs three pairs of primers to recognize the target DNA. The sequences of the primers that were designed for pathogen detection are shown in Table 1.

### Performance of the Detection System and Microfluidic Chip

**Validation of the chip.** This LAMP-based microfluidic chip had two essential components for determining the effectiveness and efficiency of the reaction: the valve and the PTC heater. The LAMP assay needs to be performed at 65°C so that the reaction solution expands, causing the liquid to flow backward or into the reaction chamber. Therefore, a valve was incorporated into the chip to prevent the reaction liquid from refluxing. First, the channel of the chip was filled with a magenta solution to evaluate the ability of the valve to seal any leaks (Fig. 2B); when the plug was pressed down, the liquid did not flow through the channel. Additionally, a 0.1 M KCl solution was utilized to facilitate conduction (Fig. 2C), allowing the voltage to decrease from approximately 3.3 V to almost 0 V when the valve was closed. This experiment demonstrated that the valve successfully regulated fluid flow.

**Fig. 5:**
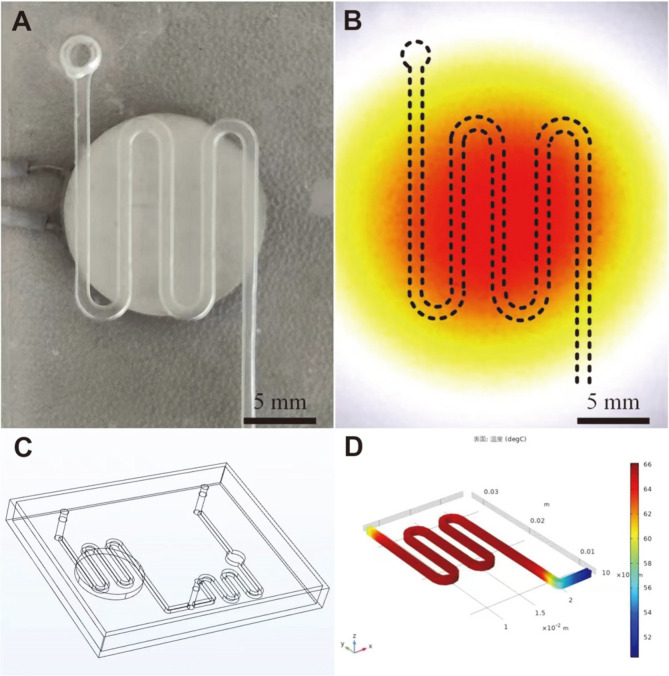
Heating effect of the PTC ceramic heater in the microfluidic chip. (**A**) photograph of the PTC ceramic heater heating the serpentine tube. (**B**) Infrared thermal image of the system. (**C**) Finite element analysis of the microfluidic chip. (**D**) Simulation results of the heating effect of the PTC.

To assess the heating effects of the system, finite element analysis was performed to simulate the temperature distribution of the liquid in the chip. Overall, the temperature of the liquid in the serpentine tubes was evenly distributed and reached the reaction temperature; the longer the tube length and diameter were, the larger the surface area of the liquid in the serpentine tube was (Fig. 5). Therefore, a higher surface-to-volume ratio enabled a more efficient heat transfer to the LAMP mixture than could be achieved with regular microtubes, which was conducive for the rapid achievement of reaction conditions.

**Validation of the detection system.** Unlike most pathogens requiring rapid detection, *S. mutans* is ubiquitous in the oral cavity; thus, its qualitative detection alone cannot determine the risk of dental caries, but bacterial quantification is essential for its early diagnosis and prevention. As LAMP assays can quickly produce a large number of reaction products and enable visual detection, a programmed OpenMV camera was used herein to segment, identify, and calculate the Lab average value of the images and quantify the experimental results.

OpenMV is a microcamera embedded in a microcontroller, which has a small volume, low power consumption, and image processing function, and can be programmed using high-level Python scripts, making it convenient to manage the complex outputs of machine vision algorithms and high-level data structures. The OpenMV camera was initially programmed in Python to transform the light wavelength and intensity of the image captured by the camera into digital signals that the computer could recognize. The digital signal was then sent to a computer, which then processed the outcomes. In this study, the average Lab value was used as a quantitative measure of the experimental results. The Lab color model was composed of brightness (L*) and two-color channels (a* and b*). Channel a* contained three colors: red for high brightness, gray for medium brightness, and dark green for low brightness. Additionally, there were three hues in channel b*: blue for low brightness, gray for medium brightness, and yellow for high brightness [31]. The Lab color mode can lessen the impact of brightness on image recognition compared with the commonly used RGB mode or grayscale value, resulting in a better image processing outcome [32]. Hence, the OpenMV camera performed quantitative analysis of the experimental results through color image recognition generated by the LAMP reaction and successfully built a visual detection platform, laying foundations for rapid *S. mutans* detection.

### Comparative Performance of the Benchtop LAMP and PCR

**Fig. 6:**
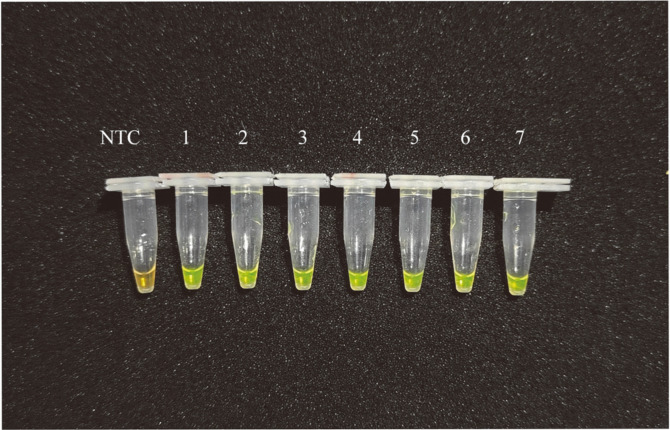
LAMP results. NTC: non-target control; 1–7: *S. mutans* samples with varying concentrations (2.2 × 10^0^–2.2 × 10^6^ CFU/ml).

**Fig. 7:**
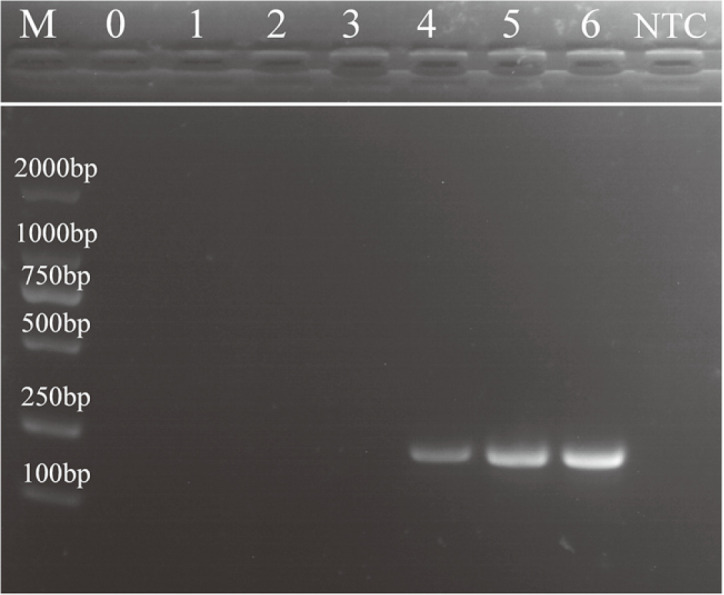
LOD of the PCR of specific regions of the *S. mutans* spaP gene using LAMP primers. Lanes 0–6: DNA of *S. mutans* at different concentrations (2.2 × 10^0^–2.2 × 10^6^ CFU/ml); lane M: 500-base pair DNA marker ladder; NTC, nontarget control.

The LAMP assay allows colorimetric detection of the target output, with the color of samples changing from yellow to green. Calcein is a metal indicator. By adding manganese ion and calcein to LAMP system, it was found that the colorimetric change of metal indicator could monitor the process of LAMP reaction. When calcein combines with Mn^2+^, there is a quenching effect on the chelating dye. With the synthesis of a large number of DNA double strands, pyrophosphate ion byproducts produced in the reaction system make Mn^2+^ combine with phosphoric acid, calcein is released to complex free magnesium, resulting in bright green color. In visible light, a color change from orange to yellow-green can also be observed by the naked eye after successful amplification [33]. In the present study, 10-fold serial dilutions of *S. mutans* were used to determine the sensitivity of the LAMP assay (Fig. 6), with the reaction products in the tubes containing *S. mutans* and a no-target control (NTC) being green and yellow, respectively, indicating that the LAMP assay could detect as few bacteria as 2.2 CFU/ml. The amplification products of LAMP and PCR were subjected to gel electrophoresis for limit of detection (LOD) comparison. Overall, the LAMP assay reached a LOD of 2.2 × 10^0^ CFU/ml, whereas that of PCR was 2.2 × 10^5^ CFU/ml (The lane 4 in the Fig. 7 is not clear enough.) (Fig. 7). Furthermore, LAMP required less than 50 min from start to result determination, whereas PCR took more than 120 min. PCR is usually performed by well-trained personnel and requires sophisticated instruments that are rarely available in outpatient departments, primary health units, or resource-limited regions and countries [34]. As PCR requires several temperature-controlled cycles, the process is relatively time-consuming and tedious. In contrast, LAMP requires only a water bath; thus, less instrumentation and energy is necessary as compared with that of the PCR approach [35]. Therefore, compared with PCR, LAMP has several advantages, such as higher sensitivity, better specificity, time- and reagent-saving, ease of operation, and lack of sophisticated equipment, thereby effectively avoiding the shortcomings of PCR [36]. In summary, the present findings demonstrate that LAMP can be used by non-technology-intensive research institutions for the general detection of organisms causing caries.

**Fig. 8:**
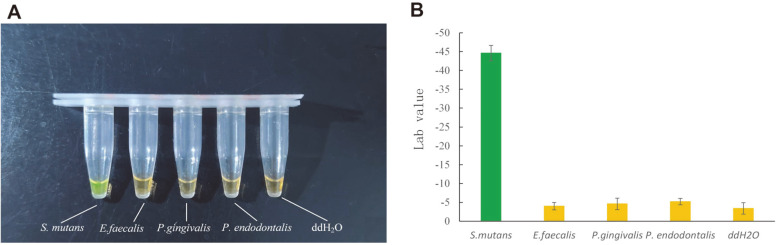
Specificity of the LAMP method. Samples containing *S. mutans*, *P. gingivalis*, *P. endodontalis*, *E. faecalis*, and ddH_2_O were tested. (**A**) Visual results. (**B**) Average L*a*b* quantification of the LAMP results using the detection system. Since the Lab value varies for different color measurements, different colors were used in the quantization table. Error bars represent the standard deviation of triplicate experiments.

Next, three common oral bacteria were used in the LAMP assay. Specificity tests revealed that after 40 min of reaction, no color change was observed in these bacteria (Fig. 8), demonstrating the specificity of the target DNA and primers. Because the three pairs of primers are required to hybridize with multiple regions of the target nucleic acid in the LAMP reaction, it has relatively high specificity [16, 37], which can prevent the influence of other common oral bacteria and achieve effective detection of *S. mutans*.

### Performance of the LAMP-Based Microfluidic Detection Platform

**Fig. 9:**
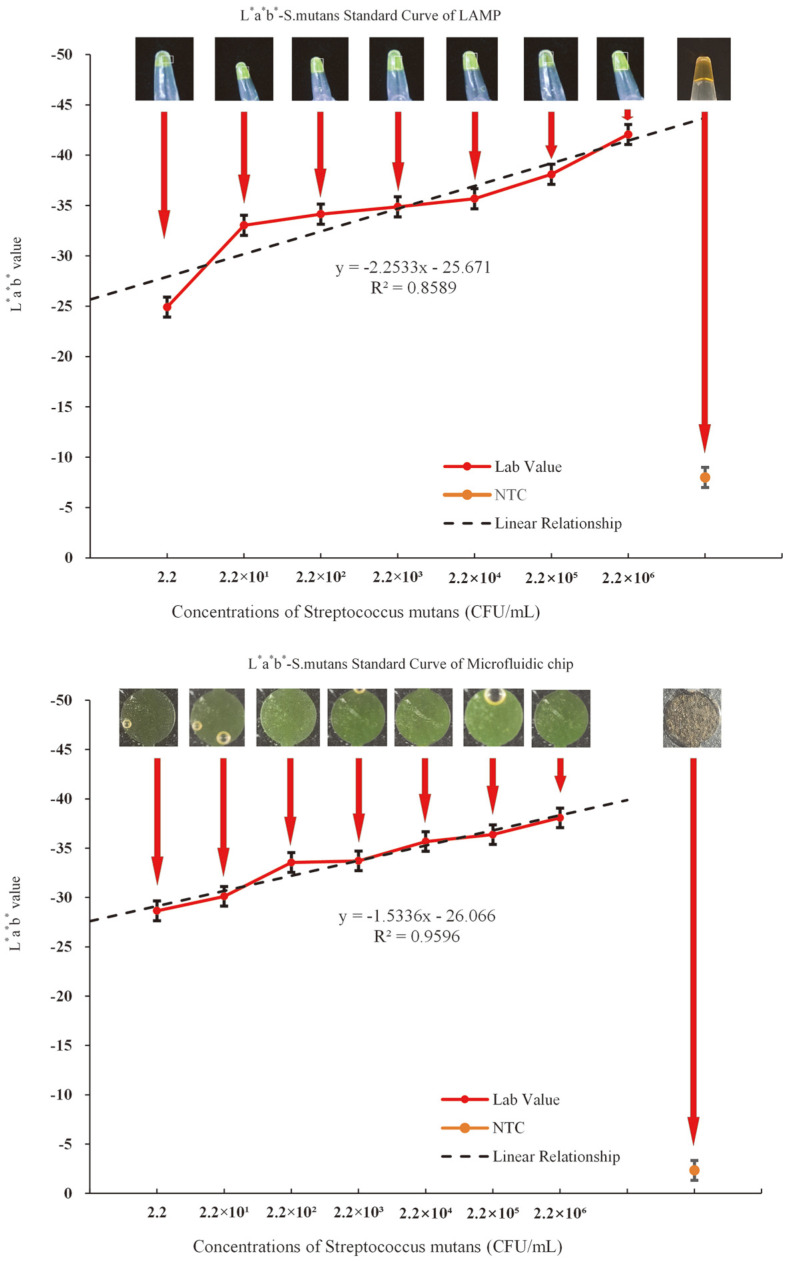
A LAMP amplification curve of *S. mutans* using a benchtop system. Error bars represent the standard deviation of the triplicates. B LAMP amplification curve of *S. mutans* using the microfluidic chip. The error bars represent the standard deviation of triplicate experiments.

Pathogen detection was performed using the microfluidic chip, and the results showed that the reaction products at various concentrations exhibited a green color, indicating that the LOD of the chip also reached 10^0^ CFU/ml. To further investigate the relationship between the bacteria concentration and the color of the amplification products, the results of the benchtop LAMP and LAMP-based microfluidic chips were analyzed. Analysis of the L*a*b* values revealed that they increased with increasing bacterial concentration in both the LAMP assay (Fig. 9A) and microfluidic chip (Fig. 9B). The results showed a good linear relationship between L*a*b* values and bacterial concentration, which was better in the microfluidic chip than in the LAMP assay (*R*^2^ = 0.9596 vs. 0.8589, respectively). The probable reason for this difference may be due to the shape of the chip chamber, which is flatter than the tube, thereby allowing the color to distribute more evenly and facilitate detection. This linear relationship proved that this microfluidic system could effectively mimic the desired experimental output. The curve for the microfluidic chip group was similar to that of the LAMP group; therefore, comparison between the two groups indicated that the LAMP-based microfluidic detection platform was capable of detecting *S. mutans* accurately. As the concentration gradient of *S. mutans* was determined according to the different colors obtained, the microfluidic chip can be used without the need for a complicated fluorescence excitation device.

This study has some limitations. First, the detection process requires bacterial lysis to be performed and does not allow for complete process detection. Second, in-chip detection requires manual valve closure and is not fully automated. Last, the detection of clinical samples is required to determine the clinical effectiveness of the system. Therefore, since the biofilm composed of mixed bacteria strains plays an important role in the formation of caries, we will use this system to detect the target pathogen in the biofilm in future work.

In conclusion, this study reports the successful development of a LAMP-based microfluidic system for the rapid and quantitative detection of *S. mutans*. This system, which integrates nucleic acid amplification, LAMP reaction, and experimental result collection and interpretation, can detect *S. mutans* at concentrations as low as 2.2 CFU/ml within 1 h. This microfluidic system may represent a promising detection method that can be used for the direct detection of multiple bacteria in saliva samples and thereby help diagnose various oral pathologies.

## Supplemental Materials

Supplementary data for this paper are available on-line only at http://jmb.or.kr.

## Figures and Tables

**Table 1:** Primers used for the LAMP assay.

Primer name	Tm (°C)	GC rate	Sequence (5′- 3′)	Target gene
SMspaP-F3	54.5	55.6%	TTAGGCAGGGTCAAGGTG	*spaP*
SMspaP-B3	51.1	42.9%	CAATTGCCATTGTAGGATCAG	*spaP*
SMspaP-FIP (F1c-F2)	69.0	50.0%	GAGTTAGGCGCATCTGAACTATCCGGACCATGTATACCAGAGCT	*spaP*
SMspaP-BIP (B1c-B2)	68.3	48.8%	GGTATGGTGCTGGTGCTATCCACAAACAAGTGTTGATGAGA	*spaP*
SMspaP-LoopF	58.1	64.7%	CCAGATCCCGGTTCGCT	*spaP*
SMspaP-LoopB	54.8	45.8%	GCTTGGTCCTAATAACAGTGTGAC	*spaP*

Tm, melting temperature
