# Supplementary material for: Rapid Detection of Streptococcus mutans Using an Integrated Microfluidic System with Loop-Mediated Isothermal Amplification
Source: J Microbiol Biotechnol. 2023 May 19;33(8):1101–10. doi: 10.4014/jmb.2304.04026 (PMC10468681; doi:10.4014/jmb.2304.04026)
Supplement: Supplementary file 1 [file jmb-33-8-1101-supple.pdf]

J. Microbiol. Biotechnol.
https://doi.org/10.4014/jmb.2304.04026

Supplementary Figure 1

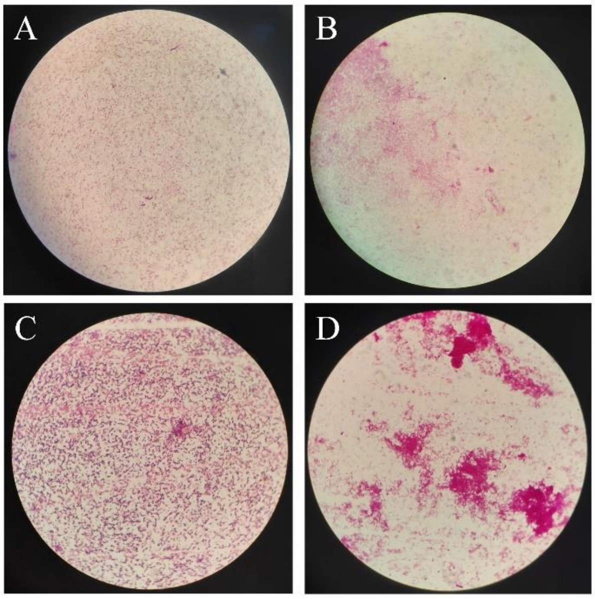

Figure S1. Gram staining observations of the four bacterial strains under a microscope(1000X). (A) *Streptococcus mutans*. (**B)** *Porphyromonas endodontalis*. (**C**) *Enterococcus faecalis*. (**D)** *Porphyromonas gingivalis.*
